# Venous intestinal ischemia of fungal origin as a cause of intestinal obstruction in immunocompromised patients: case report and literature review

**DOI:** 10.1186/s13099-024-00658-0

**Published:** 2024-11-10

**Authors:** Diana Marcela Grajales-Urrego, Fabián Mantilla-Sylvain, Mariam Carolina Rolon-Cadena, William Mauricio Basto-Borbón, Johanna Álvarez-Figueroa

**Affiliations:** 1 Department of Pathology and Laboratories, Fundación Santa Fe de Bogotá, Bogotá, D.C Colombia; 2 Department of Surgery, Faculty of Medicine, Universidad El Bosque, Bogotá, D.C Colombia; 3 Department of Surgery, Faculty of Medicine, Fundación Santa Fe de Bogotá, Bogotá, D.C Colombia; 4 School of Medicine, Universidad de los Andes, Bogotá, D.C Colombia

**Keywords:** Mucormycosis, Gastrointestinal mucormycosis, Human immunodeficiency virus, Acute myeloid leukemia, Intestinal ischemia, Venous thrombosis.

## Abstract

**Background:**

Mucormycosis is a highly lethal opportunistic fungal disease caused by ubiquitous molds of the order Mucorales, with Rhizopus, Lichtheimia and Mucor being the most common genera. This rare disease primarily affects immunocompromised patients, with presentations ranging from rhino-orbito-cerebral infections to disseminated mucormycosis with angioinvasion, leading to thrombosis and tissue infarction. Gastrointestinal mucormycosis is the least common clinical presentation and is believed to be secondary to spore ingestion. It can involve multiple components of the gastrointestinal tract, such as the stomach, liver, ileum, and colon, with nonspecific manifestations, including pain, nausea, vomiting, and abdominal distension. The initial clinical presentation may even manifest as gastrointestinal bleeding due to gastric ulceration or intestinal perforation.

**Case presentation:**

Here we present the case of a 48-year-old male patient with a 9-year history of human immunodeficiency virus (HIV) infection who was hospitalized in the context of febrile neutropenia and whose acute respiratory infection was documented; therefore, antibiotic treatment was initiated. However, due to persistent febrile peaks and peripheral blood showing documentation of multilineage cytopenias, a bone marrow biopsy was performed, compatible with presenting features of marrow myelodysplasia. During hospitalization, the patient presented left flank abdominal pain, and an abdominal computed tomography (CT) scan revealed signs of intussusception of a small bowel loop at the distal jejunum level, leading to intestinal obstruction with ischemic progression, requiring ileectomy (60 cm). Histopathological analysis of the resected intestine revealed severe transmural ischemic changes associated with venous thrombosis due to fungal structures, with histochemical studies demonstrating the presence of zygomycete (Mucor) fungal structures, leading to the initiation of treatment with amphotericin B. However, despite treatment, the patient experienced progressive clinical deterioration with persistent fever and ventilatory failure, with follow-up tests showing absolute neutropenia and blood cultures positive for yeast, leading to death 52 days after admission.

**Conclusions:**

The diagnosis of intestinal mucormycosis may be delayed due to the lack of specificity of the signs and symptoms. Pathologists as well as histopathological studies are essential for timely treatment.

## Background

Invasive fungal infections are important causes of morbidity and mortality in immunocompromised patients. In HIV infection, zygomycosis has been reported more rarely since it occurs more often in diabetic patients or patients with malignant neoplasms. Furthermore, the use of antiretrovirals radically decreased fungal infections in these patients. However, it is important to highlight that the use of antiretrovirals can predispose patients with HIV infection to diabetes and malignant neoplasms due to morphological alterations at the bone marrow level, increasing the risk of fungal infections such as mucormycosis [1, 2]. On the other hand, patients undergoing chemotherapy regimens for acute leukemia are generally diagnosed with fungal infections through the neutropenic phase during the course of treatment, frequently presenting in the context of febrile neutropenia. Unfortunately, these infections are fatal because they do not respond to antibiotic therapy or initial standard empirical antifungal agents [1]. 

A high level of suspicion in patients with leukemia and fever during chemotherapy is of critical importance to achieve an early diagnosis, leading to early initiation of antifungal treatment. Notably, zygomycosis can occasionally occur at anatomical sites far from the lungs or paranasal sinuses, which are the most frequent sites [1, 3]. Furthermore, the clinical symptoms and signs are nonspecific, as is the case of the patient we present below, where an infection due to many mycoses occurs in an unusual form such as gastrointestinal mucormycosis. Anatomopathological analysis revealed infiltration by the fungus at the wall of the blood vessels at the venous level, which made this presentation even rarer, with the presence of thrombosis followed by ischemic infarction and intestinal necrosis [3]. 

## Case presentation

A 48-year-old male patient was admitted to the emergency department due to fever, cough with scant no purulent mucoid expectoration, headache, and changes in mood. It is important to consider that the patient had a medical history of human immunodeficiency virus (HIV) infection managed with antiretroviral therapy (Efaviricenz + emtricitabine + tenofovir disoproxil fumarate) for 9 years with a recent (< 1 month) undetectable viral load and a CD4 + lymphocyte count of 729. Broad-spectrum antibiotic management was initiated, extensive imaging studies were requested (chest X-ray, hepatobiliary ultrasound, computed tomography of the skull, neck, thorax and total abdomen) within normal limits, as well as a negative infectious profile (Cytomegalovirus: IgG 279, nonreactive IgM, HBV Ab, HBsAg, HCV Ab, *Treponema pallidum*, nonreactive *Mycobacterium tuberculosis* and PCR COVID-19 not detected, EBV: IgG 2080 U/µL). Laboratory analyses revealed a white blood cell count of 1.73 × 10^3^/µL, an absolute neutrophil count of 0.68 × 10^3^/µL (39.2%), a lymphocyte count of 0.74 × 10^3^/µL, a platelet count of 18 × 10^3^/µL, a hemoglobin level of 8.8 g/dL and a hematocrit of 25.5%. Aerobic and anaerobic blood cultures as well as fungi cultures were negative. Similarly, there was no detection of *Mycobacterium tuberculosis* through GeneXpert Ultra. Due to the findings of pancytopenia, the patient was evaluated by the hematology service, who considered performing a bone marrow biopsy consistent with myelodysplastic neoplasia. Flow cytometry revealed increased myeloblasts (15.5%) and a karyotype with 11% myeloblasts and plasma cells. A total of 2%, 15%, 69%, 2% and 1% of lymphocytes, myelocytes, monocytes and erythroid cells, respectively, were diagnosed with acute myeloid leukemia (AML), which required hospitalization for management, symptomatic control of the disease through chemotherapy, and the initiation of valganciclovir due to cytomegalovirus reactivation (356 copies).

**Fig. 1:**
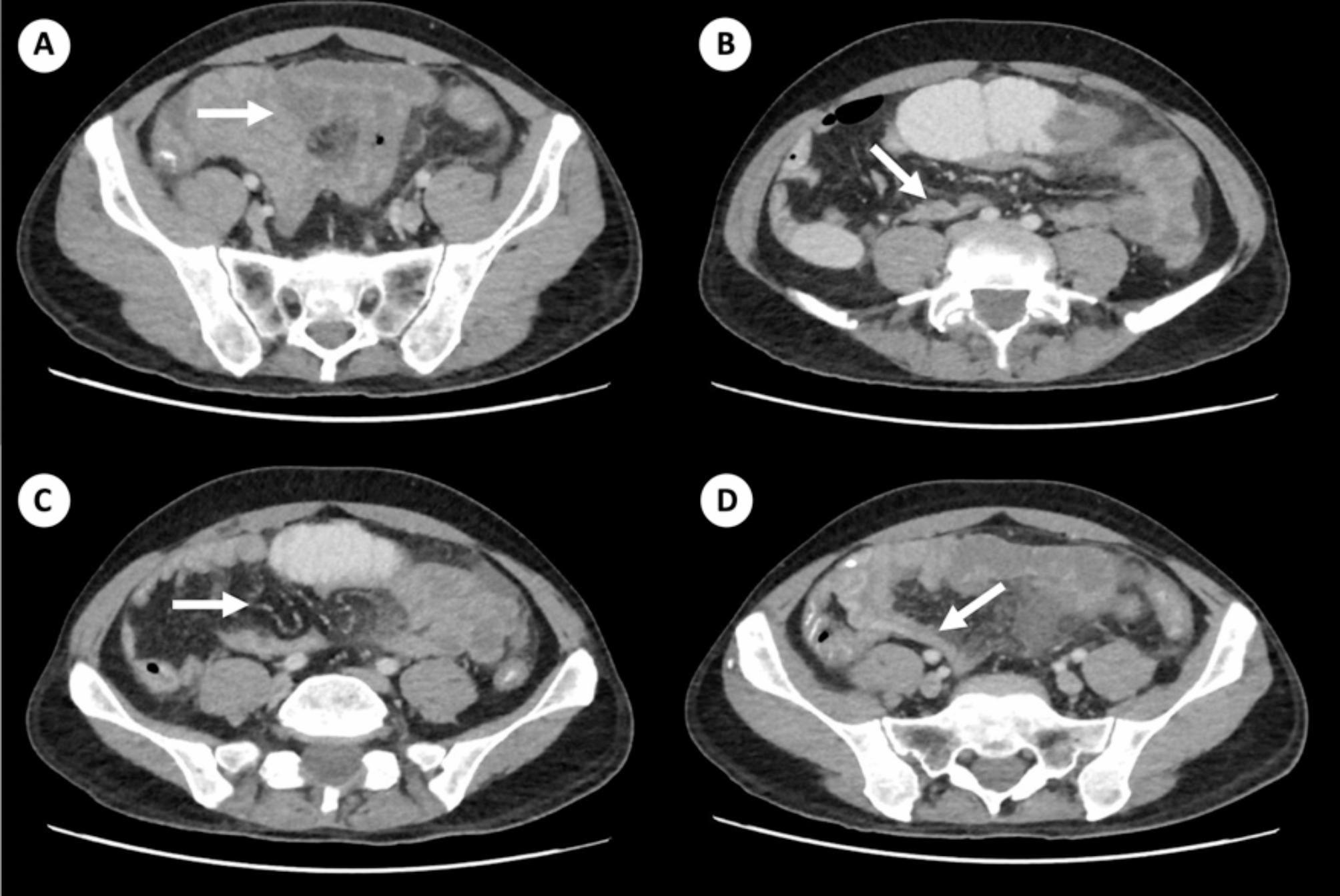
Thin loop intussusception signs in the distal jejunum to the left that condition intestinal obstruction associated with signs of loop suffering in this location and of the adjacent small intestinal loops as well as the colon falling. **A**, Distention of small intestinal loops with retrograde distension and wall edema. **B**, Presence of retrograde distention of loops with anterograde collapse with “sausage like” sign noted. **C**, Even clearer sign in the circle. **D**, Mesenteric edema associated with venous congestion

During his hospital stay, the patient presented for three days with distension and abdominal pain located in the left flank, so colitis was suspected; metronidazole was started for coverage. However, the patient experienced persistent abdominal pain, so a new CT scan of the abdomen was requested, where thin loop intussusception was reported in the distal jejunum, causing intestinal obstruction (Fig. 1). Due to these findings, exploratory laparotomy was performed where ischemia was found in patches of thin loops with thickened mesentery and clots at the venous level, suggesting venous thrombosis. Consequently, ileotomy (60 cm of the intestinal ischemia) and partial omentectomy were performed; spun cores and a negative pressure system were scheduled for new surgical review within 48 h. During the reintervention, ischemic involvement of the small intestine of approximately 20 cm was again observed, so it was resected, an end-to-side anastomosis was performed using the Barcelona-type jejunum technique, and the negative pressure system continued.

**Fig. 2:**
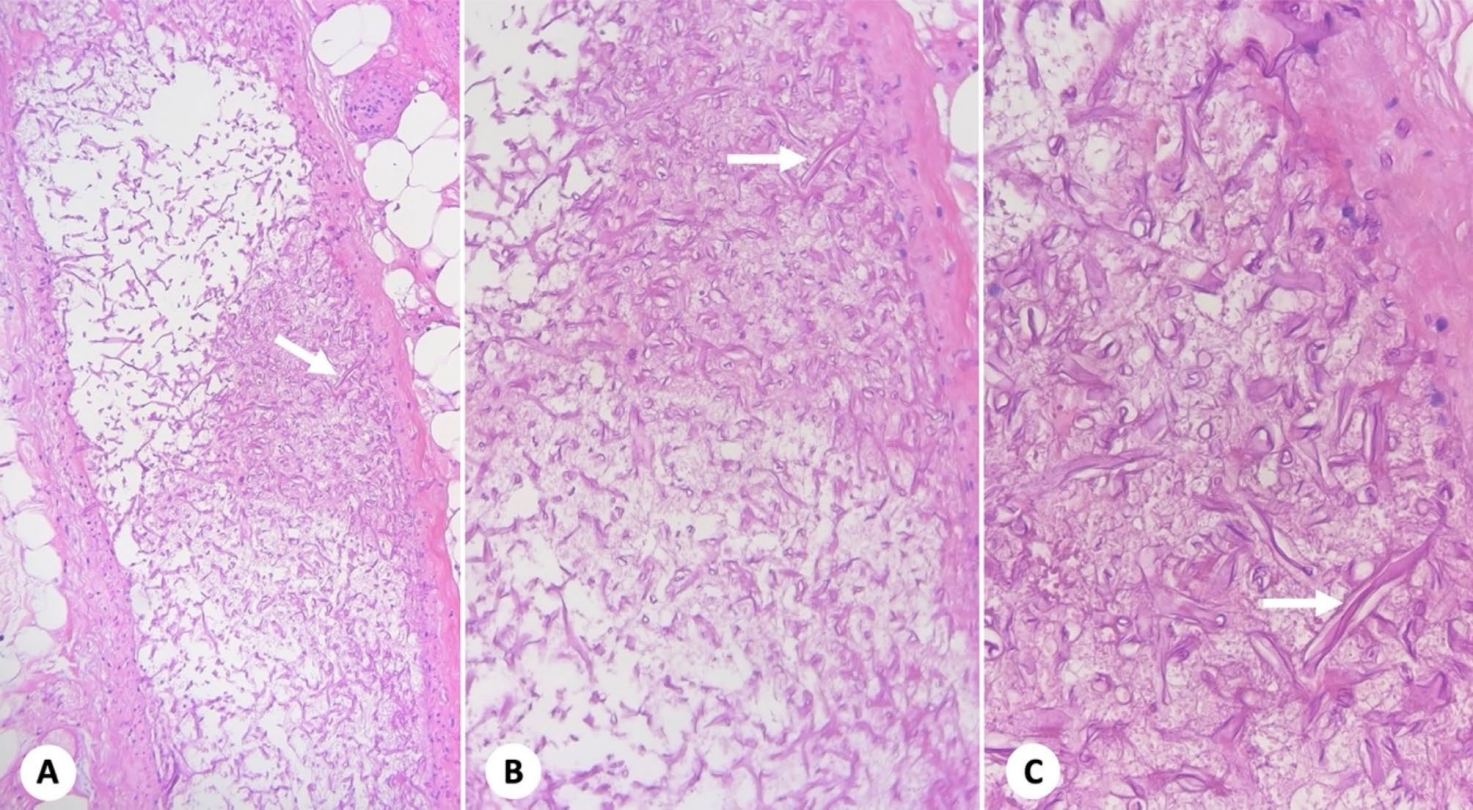
Fungal structures compatible with Mucor in venous structure (**A**, 10X. **B**, 20X. **C**, 40X; H&E)

**Fig. 3:**
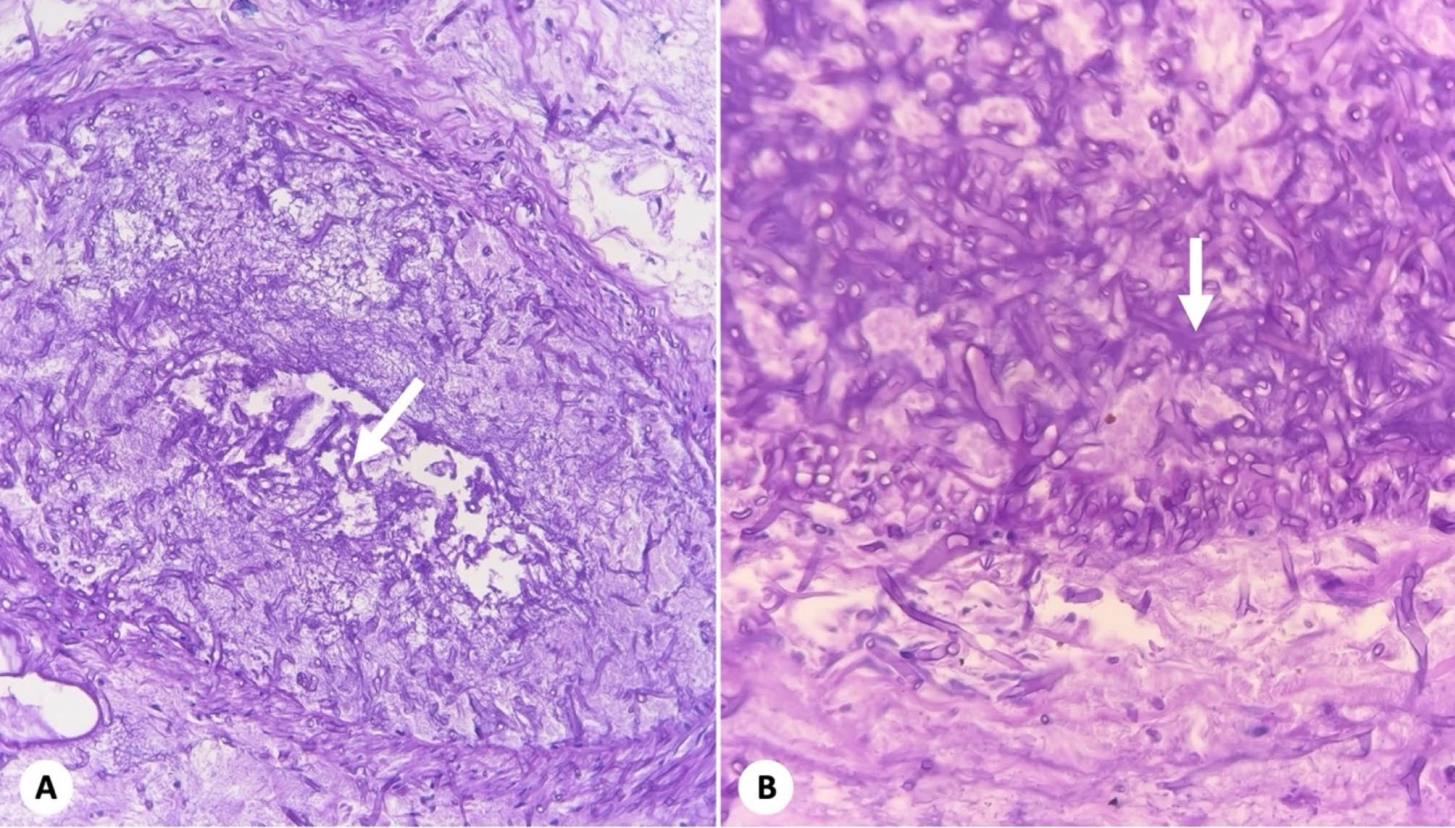
Fungal structures compatible with Mucor in venous structures. **A**, Periodic Acid-Schiff with diastase staining (PAS-D; 40X). **B**, Non-septate hyphae (H&E; 40X)

The patient was admitted to the intensive care unit (ICU) due to clinical deterioration, persistent fever and increased breathing. Blood analysis of the control paraclinical test revealed absolute neutropenia, anemia and thrombocytopenia, so broad-spectrum antibiotic therapy was started. Control blood cultures revealed the presence of yeast. Histopathology of the small intestine (Fig. 2) revealed acute inflammation, transmural necrosis and venous vascular invasion with numerous wide, irregular, branched and non-septate hyphae, consistent with Mucor-type fungi. Periodic acid-Schiff with diastase (PAS-D) and Gomori stains confirmed the presence of hyphae with thick focal branches, thin walls, rare septation, and occasional bulbous dilation, which are typical mucor types (Fig. 3). Therefore, management with amphotericin B was initiated, but the patient continued to have an unfavorable condition, with persistent febrile peaks, requiring ventilatory and inotropic support. A lack of response to medical treatment led to the patient’s death 52 days after admission to the hospital.

## Discussion and conclusions

Mucormycosis is an angioinvasive opportunistic fungal infection caused by fungi of the order Mucorales. Rhizopus species are the most prevalent Mucorales and account for more than 70% of infections [4]. Its incidence cannot be precisely measured due to limited population studies, but various studies have shown an increase in its occurrence which account for 1.7 cases per 1 million individuals [4, 5]. The most significant predisposing conditions for mucormycosis include diabetes mellitus (DM), with or without ketoacidosis, hematological neoplasms, other malignant neoplasms, transplants, prolonged neutropenia, corticosteroids, trauma, iron overload, illicit intravenous drug use, neonatal prematurity, and malnutrition [4]. Immunocompetent patients can also be affected when fungal spores are directly inoculated into the skin due to trauma or burn, facilitated by the fungus’s cell wall composition and genetic alterations, allowing rapid growth in the host environment and evasion of its defenses [5]. 

Cases of mucormycosis in HIV + individuals without any additional risk factors such as the case presented have rarely been reported [6], owing to the initial response of neutrophils rather than lymphocytes to fungal infections [2, 7]. The majority of HIV patients who present with mucormycosis have associated conditions (e.g. DM) [2]. In some cases, this may be related to the use of the latest generation of antiretroviral regimens that can predispose patients to diabetes and/or the appearance of malignant neoplasms [2] such as our previously reported case, who was diagnosed with AML [8]. Therefore, mucormycosis may become more common in association with HIV infection in patients with severe neutropenia and in those who lack phagocytic function, increasing their susceptibility to fungal infections [9]. Phagocytes play a critical role in the host defense against Mucorales, as macrophages phagocyte inhaled sporangiospores, preventing their germination, while neutrophiles produce oxidative metabolites and cationic peptides, killing the fungus [10]. For instance, an important prognostic factor in patients with hematological malignancies in the recovery from neutropenia [10]. 

These pathogens can infect various organs, including the head and neck, nasal cavity, lungs, skin, central nervous system, and gastrointestinal (GI) tract, causing infarction and tissue necrosis. This is mediated through the expression of spore coat protein homologue (CotH) proteins in spores and hyphae, facilitating adhesion to endothelial cell receptors, angioinvasion, and dissemination [11]. The hyphae of Mucorales specifically recognize the host receptor GRP78 on endothelial cells, an interaction mediated by the fungal ligand CotH3 for endocytosis of the fungus [12]. Angioinvasion by Mucorales causes endothelial injury and death leading to thrombosis, dissemination, and tissue necrosis [12]. The toxic metabolites produced by Mucorales (e.g., mucoricin) have shown to induce inflammation, vascular permeability, and tissue necrosis [12]. The production of lytic enzymes and proteases, along with mycotoxins, enhances fungal invasion. Therefore, the main pathological feature in affected organs is angioinvasion, which results in hemorrhage, infarction, and suppurative inflammation [7, 11]. 

Gastrointestinal mucormycosis is a relatively rare manifestation of infection, with a mortality rate between 40% and 85%. It is most frequently observed in the stomach (57.5%), followed by the small intestine (10.3%) and the colon (32.2%), as observed in this case [13]. Mucorales primarily enter the gastrointestinal tract through nasogastric intubation, infected wooden tongue depressors, and contaminated foods such as fermented milk, dried bread products, and alcoholic beverages derived from infected corn [5, 13]. Due to the nonspecific clinical signs of gastrointestinal mucormycosis, including abdominal pain and distension, rectal bleeding, and fever, the disease is often diagnosed late. The lack of differentiation from other gastrointestinal diseases complicates its diagnosis, which underscores the need for a high index of suspicion [13, 14]. 

Our patient presented with angioinvasion due to mucormycosis. This complication has been previously reported as potentially fatal due to the fungus’s strong tendency to invade blood vessels, leading to necrosis, thrombosis and tissue infraction, causing intestinal necrosis with visceral perforation, peritonitis and massive gastrointestinal bleeding [2]. Prominent infarcts and angioinvasion characterize invasive disease and are predominant in neutropenic patients, which is consistent with the present case [14]. 

Histopathology and various clinical cultures are the cornerstones of the diagnosis of mucormycosis [7]. The histopathological characteristics of the affected tissue of our patient revealed the presence of extensive necrosis with numerous large, branched, pale, wide, flat, non-septate hyphae, with common high-angle bifurcations (90°). Staining with hematoxylin-eosin made it possible to evaluate neutrophilic or granulomatous inflammation and the presence of fungal elements, which in some cases could only show the cell wall of the fungus without structures inside or, sometimes, very degenerated hyphae. The dyes used to visualize fungal walls include Grocott’s methenamine silver (GMS) and periodic acid-Schiff (PAS). PAS provided us with better visualization of the surrounding tissue than GMS, allowing for more detailed analysis of the microanatomy of the fungus [5, 14]. 

Treatment for mucormycosis typically involves surgical debridement of the involved tissues and antifungal therapy, along with a suggested empirical antifungal therapy, considering the adversity of infection and the risk of poor outcomes [15]. Treatment for the present case involved surgical removal of infected tissue and debridement of necrotic tissue to prevent the progression of infection to vital structures or spread to other sites. Adequate surgical debridement of compromised tissues can improve the penetration of antifungal agents into the still viable infected tissue associated with the early initiation of antifungal therapy, when mucormycosis infection is suspected [11, 14]. However, the patient had severe neutropenia, associated with anemia and thrombocytopenia, in addition to a systemic mycosis; despite the initiation of broad-spectrum antifungal and surgical procedures, the patient continued with an unfavorable evolution without response to medical treatment.

Early diagnosis and treatment are crucial for preventing complications and death, such as the case described above, which had a fatal outcome. However, initial diagnosis is challenging due to the non-specificity of signs and symptoms and the lack of a diagnostic tool to confirm the disease. It is important to highlight the role of pathologist and the histopathological studies, which are essential for diagnosis and treatment.

## Data Availability

No datasets were generated or analysed during the current study.

## Abbreviations

AML: Acute myeloid leukemia
COVID-19: Coronavirus disease
COTH: Spore coat protein homologue
CT: Computed tomography
DM: Diabetes mellitus
EBV: Epstein-Barr virus
GI: Gastrointestinal
GMS: Grocott methenamine silver
H&E: Hematoxylin and eosin
HBsAg: Hepatitis B surface antigen
HBV Ab: Hepatitis B Virus antibody
HCV Ab: Hepatitis C Virus antibody
HIV: Human immunodeficiency virus
ICU: Intensive Care Unit
IgG: Immunoglobulin G
IgM: Immunoglobulin M
PAS: Periodic acid-Schiff
PCR: Polymerase chain reaction
